# Supplementary material for: Low-Energy Extracorporeal Shock Wave Ameliorates Streptozotocin Induced Diabetes and Promotes Pancreatic Beta Cells Regeneration in a Rat Model
Source: Int J Mol Sci. 2019 Oct 5;20(19):4934. doi: 10.3390/ijms20194934 (PMC6801760; doi:10.3390/ijms20194934)

Supplemental Information

Low-energy Extracorporeal Shock Wave Ameliorates Streptozotocin induced diabetes and Promotes Pancreatic Beta Cells Regeneration in Rat Model

Chang-Chun Hsiao, Cheng-Chan Lin, You-Syuan Hou, Jih-Yang Ko, and Ching-Jen Wang

FIGURE LEGENDS

Supplemental Figure 1. Illustration of a low-energy extracorporeal shock wave (SW) experiment set-up. (a) Using ultrasound probe and ultrasound system localizes for pancreas position. (b) Ultrasound pancreas image. (c) Low-energy SW treatment zone along the pancreas. (d) ultrasound system. (e) Low-energy SW system.

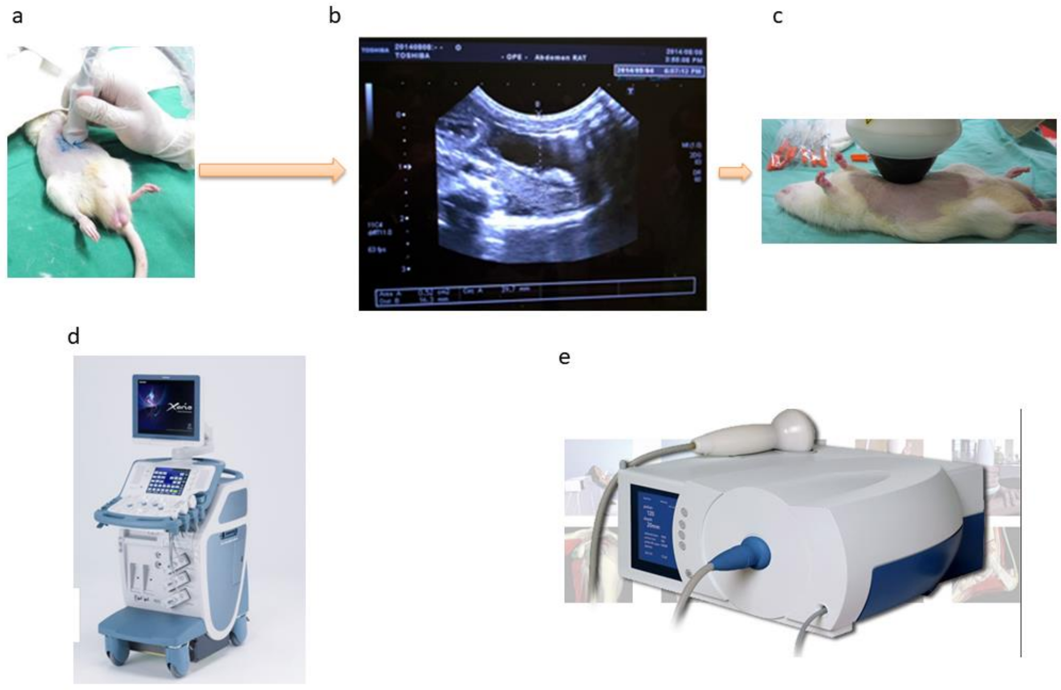

Supplement: Supplementary file 1 [file ijms-20-04934-s001.pdf]
